# Lipogenesis in arterial wall and vascular smooth muscular cells: regulation and abnormalities in insulin-resistance

**DOI:** 10.1186/1475-2840-8-64

**Published:** 2009-12-23

**Authors:** Nadjiba Hamlat, Fabien Forcheron, Samia Negazzi, Peggy del Carmine, Patrick Feugier, Giampiero Bricca, Souhila Aouichat-Bouguerra, Michel Beylot

**Affiliations:** 1 ERI-22 - EA4173, Faculté Rockefeller, UCBLyon1, Lyon, France; 2 USTB H Boumedienne, Alger, Algérie; 3 ANIPHY, Faculté Rockefeller, UCBLyon1, Lyon, France; 4 Hospices Civils de Lyon, Lyon, France

## Abstract

**Background:**

Vascular smooth muscular cells (VSMC) express lipogenic genes. Therefore *in situ *lipogenesis could provide fatty acids for triglycerides synthesis and cholesterol esterification and contribute to lipid accumulation in arterial wall with aging and during atheroma.

**Methods:**

We investigated expression of lipogenic genes in human and rat arterial walls, its regulation in cultured VSMC and determined if it is modified during insulin-resistance and diabetes, situations with increased risk for atheroma.

**Results:**

Zucker obese (ZO) and diabetic (ZDF) rats accumulated more triglycerides in their aortas than their respective control rats, and this triglycerides content increased with age in ZDF and control rats. However the expression in aortas of lipogenic genes, or of genes involved in fatty acids uptake, was not higher in ZDF and ZO rats and did not increase with age. Expression of lipogenesis-related genes was not increased in human arterial wall (carotid endarterectomy) of diabetic compared to non-diabetic patients. *In vitro*, glucose and adipogenic medium (ADM) stimulated moderately the expression and activity of lipogenesis in VSMC from control rats. LXR agonists, but not PXR agonist, stimulated also lipogenesis in VSMC but not in arterial wall *in vivo*. Lipogenic genes expression was lower in VSMC from ZO rats and not stimulated by glucose or ADM.

**Conclusion:**

Lipogenic genes are expressed in arterial wall and VSMC; this expression is stimulated (VSMC) by glucose, ADM and LXR agonists. During insulin-resistance and diabetes, this expression is not increased and resists to the actions of glucose and ADM. It is unlikely that this metabolic pathway contribute to lipid accumulation of arterial wall during insulin-resistance and diabetes and thus to the increased risk of atheroma observed in these situations.

## Introduction

Excessive accumulation of lipid substrates in non-adipose tissues has serious adverse effects on cell functions (lipotoxicity) [1] and can contribute to the development of insulin-resistance [2]. Indeed numerous studies have shown an inverse relationship between tissue lipid accumulation and sensitivity to insulin of glucose metabolism in liver [3] and skeletal muscle [4]. In such studies tissue lipid accumulation is usually appreciated by measuring triglycerides (TG) content although recent studies clearly show that deleterious effects are not due to the accumulation of TG itself [5] but to other lipid metabolites such as palmitate, diacylglycerols and ceramide [6-8]. The arterial wall of obese Zucker rats is insulin-resistant although this resistance is limited to the PI3-kinase pathway [9]. In addition, this pathway has also been found insulin-resistant in cultured vascular smooth muscular cells (VSMC) of diabetic patients [10]. The mechanisms responsible for this resistance have not been clarified. It could result from excessive concentration of plasma cytokines such as TNFα [11], of raised levels of angiotensin II or local overexpression of components of the renin-angiotensin system [10,12]. It could also result of excessive accumulation of lipid substrates. Actually, arterial wall accumulate TG with aging [13]. In addition, foam cells of atheroma plaques accumulate not only cholesterol but also significant amounts of TG (8-10%of total lipid) [14-16]. These cellular TG modify the physical state of stored esterified cholesterol and this could affect the way it is hydrolyzed and effluxed [17]. Therefore, accumulation of TG could also play a role in atheroma. TG synthesis, as well as cholesterol esterification, requires long chain fatty acyl-CoA. Acyl-CoA can be provided by the uptake of circulating lipids (plasma non esterified fatty acids, NEFA, or TG-fatty acids of TG rich lipoproteins) but also by *in situ *synthesis, the pathway of *de novo *lipogenesis (DNL). Indeed, arterial wall, foam cells, macrophages and vascular smooth muscular cells (VSMC) incorporate labeled acetate into phospholipids and TG [18]. More recently, Davies et al [13] showed that human VSMC express lipogenic genes such as Srebp-1c, the transcription factor mediating the lipogenic action of insulin [19], and fatty acid synthase (FAS) and that these expressions, and the intracellular accumulation of TG, are increased by culture in an adipogenic differentiation medium (ADM). Moreover, TO901317, a LXR agonist, also stimulated the expression of Srebp-1c and FAS suggesting that the lipogenic action of LXR described in liver, adipose tissue and skeletal muscle [20-23] is also present in VSMC. Lastly Davies et al found that FAS and Srebp-1c are expressed in human atherosclerotic lesions and suggested that enhanced VSMC lipogenesis and lipid accumulation could be involved in the development of atheroma [13]. This possibility should be kept in mind when developing nuclear receptor agonists for treatment of atherosclerosis.

Insulin-resistance and type 2 diabetes are risk factors for atherosclerosis and are characterized by high concentrations of insulin and/or glucose. Lipogenesis is stimulated in tissues such as liver by insulin and glucose [19,24,25]. If present in arterial wall, this stimulation could result in increased tissue lipid accumulation, aggravating further the resistance to insulin of local glucose metabolism, and possibly contribute to the accelerated atherosclerosis of insulin-resistance and diabetes. However, the expression of lipogenic genes is not increased but rather decreased in skeletal muscle of insulin-resistant and type 2 diabetic subjects, and is resistant to the action of insulin [26]. Therefore, our aims were to determine i) if TG accumulation is increased in arterial wall in experimental models of insulin-resistance and diabetes and if an increased expression of lipogenesis-related genes could contribute to this increase, ii) if lipogenesis is stimulated by insulin and glucose in VSMC and if this response is modified by insulin-resistance. In addition, we determined whether lipogenesis in VSMC is responsive or not to other hormones (thyroid hormones, angiotensin II) known to stimulate it in other cells such as hepatocytes or adipocytes. Lastly, the LXR agonist (TO90137) used by Davies et al has been shown to be actually a dual, LXR and PXR agonist [27]. PXR stimulates lipogenesis in liver [28] and is expressed in the vasculature [29]. Therefore we verified whether the effects of TO901317 in VSMC were mediated by activation of LXR or of PXR.

## Materials and methods

### In vivo studies in rats

These studies were conducted in accordance with the French regulation for experimentation in animals. Male Zucker obese (ZO, n = 18) and Zucker diabetic (ZDF, n = 15) (fa/fa) rats and their control, normal littermates (controls CO, n = 18, and CZ, n = 15, respectively, +/+) (Charles River, L'Arbresle, France) were housed at arrival (six weeks old) in an animal facility with controlled temperature (22 ± 1°C) and lightning (light on at 7:00 AM and off at 7:00 PM). Throughout the study they had free access to water and food. All ZDF rats and their controls received the diet (Purina 5008, protein 26.8%, carbohydrate 56.4% (91% starch, 9% simples carbohydrates), fat 16.7% of caloric value, IPS, London, UK) recommended for the development of diabetes in male ZDF rats. ZO rats and their controls received standard diet. Body weight was recorded once a week. After one week of acclimatizing (age of 7 weeks) five rats of the ZDF and CZ groups and six of the ZO and CO groups were sacrificed for blood collection and tissue sampling. The remaining rats were sacrificed at the age of 14 weeks or of 21 weeks (5 rats of the ZDF and CZ groups and 6 rats of the ZO and CO groups at each sacrifice)

The day of sacrifice, food was removed at 08:00 AM and rats anesthetized at 02:00 PM (pentobarbital IP 60 mg/kg), in the post-absorptive state. Blood was collected and plasma stored at -20°C until analysis. Thoracic aorta was removed, flushed with cold isotonic saline, carefully cleaned of perivascular adipose tissue and flash frozen in liquid nitrogen before storage at -80°C until analysis. Liver samples were also collected from ZO and CO rats, washed with cold isotonic saline, flash frozen with liquid nitrogen and stored at -80°C until analysis

### Carotid endarterectomy in human subjects

The procedure was approved by local ethical committee and all subjects gave their informed consent. Human atheroma plaques were removed during carotid endarterectomy from 12 subjects (8 males, 4 females, aged 65 ± 5 years). 6 subjects had overt type 2 diabetes with mild glucose control (plasma glucose: 8.91 ± 1.90 mM). The others had evidence of insulin resistance with normal plasma glucose (4.66 ± 0.30 mM) and moderate increase in basal level of plasma insulin concentration (16.8 ± 1.7 mU/L, normal <10 mU/L). 3 diabetic subjects received metformin and 3 sulfonylurea treatment. Diabetic and non-diabetic patients had comparable BMI (26.7 ± 2.0 vs 26.5 ± 1.8) but diabetic subjects had higher plasma cholesterol (5.46 ± 0.16 vs 4.40 ± 0.41 mM p < 0.05) and TG (2.28 ± 0.19 vs 1.51 ± 0.11 mM p < 0.01) levels. Samples collected directly in the surgery room were immediately divided in 2 parts, atheroma plaque and macroscopically intact tissue (MIT) situated in the vicinity of the plaque, and were flash frozen in liquid nitrogen [30].

### In vitro studies

#### Vascular smooth muscle cells culture

Explants were obtained from thoracic aorta [30] of ZO and CO rats (14 weeks old); they were prepared after removing adventitia by collagenase action (0.1%, type IA, Sigma, L'Isle d'Abeau, France). Small fragments were prepared and placed in 25 cm^2 ^culture dishes in VSMC culture medium (Promocell, Heidelberg, Germany) and maintained at 37°C under air-CO_2 _(95%-5%) atmosphere until they reached confluence. Then, VSMC were trypsinized (0.08% of trypsin; Gibco, USA) and subcultured. For the experiments, 10^6 ^VSMC/ml/well were seeded in 6 well plates in their usual medium for at least two days. Twenty four hours before starting the experiments, culture medium was replaced by a basal VSMC medium without fetal calf serum. On the first day of the experiments cells were collected in two wells (duplicate) for basal values (D0); culture medium was replaced in other wells and the test substances were added at appropriate concentrations. All experiments were done with cells at passage 3 to 5 or earlier.

#### Effects of glucose and adipogenic differentiation factors (ADM)

Cells were cultured for 3, 7 or 21 days [13] in the presence of basal (1 g/l) or high (5 g/l) glucose concentration and in the presence or absence of ADM (insulin 1.2 μM, dexamethasone 100 nM, tritiodothyronine (T3) 1 nM and 3 isobutyl-1-methyl xanthine (IBMX) 0.25 mM, final concentrations in culture medium). This adipogenic differentiation medium is identical to the one shown by Davies et al to stimulate the expression of lipogenic genes in human VSMC [13]. Cells remained viable throughout the experiments. We tested also the effects of the addition (during 3 days and 7 days) of insulin or T3 alone (same final concentrations). Lastly, since components of the renin-angiotensin system are expressed in arterial wall and VSMC [10] and angiotensin II (AngII) stimulates adipocyte differentiation as well as FAS expression and lipogenesis in adipose cells and liver [31,32], we also tested the effects of AngII (100 nM) on the expression of lipogenic genes. In order to determine if modifications of the expression of lipogenic genes is accompanied by parallel modifications of the activity of the lipogenic pathway we measured in most experiments this activity by determining the incorporation of deuterium from deuterated water into the palmitate of cellular TG [28]. In short deuterated water (30 μl/ml of culture medium) was added 24 h before the end of experiments. 24 h later culture medium and VSMC were collected for measures of deuterium enrichment.

### Effects of LXR and PXR agonists

The dual LXR and PXR agonist TO901317 was from Calbiochem (Merck, Darmstadt, Germany), others, specific, LXR agonists (GW3965, paxillin [27,28,33]) and the PXR agonist (Pregnen-3B-OL-20-ONE-16A-Carbonitrile, PCN) were from Sigma. Compounds were dissolved in ethanol (10-30 mM stock solution) and used at the final concentrations of 10 μM (TO901317 and paxillin) and 30 μM (PCN). Cells obtained from aortas of CO rats were cultured in the absence (control) or presence of one of the agonists during three days. Ethanol was added in the control culture at the same final concentration than in the test cultures. GW3965 was given by oral gavage to mice (40 mg/kg in 0.5% methylcellulose once a day during three days, n = 12). Control mice (n = 12) received only methylcellulose. Thereafter plasma was sampled and mice were sacrificed for collection of liver and aortas. Six aortas from each group were used for determination of TG content and six for mRNA measurements.

### Determinations

Blood glucose levels were measured with a glucometer (One Touch Ultra, Life technology, Issy-Les-Moulineaux, France), plasma and tissues TG by enzymatic methods [34] and insulin by ELISA (Cristal Chem, Downers Grove, Il, USA). For measurement of aortic TG concentrations, parts of the aortas were homogenized in chloroform/methanol (1:2, v:v). The chloroform phase was collected, washed with water and dried under nitrogen. Extracted lipids were dissolved in propanol for enzymatic determination of TG concentration [35]. The same procedure was used for determination of TG concentrations in VSMC. Measurements of deuterium enrichments in the palmitate of VSMC TG were performed as previously described [28,36,37] as well as the calculation of the contribution of lipogenesis to the cellular TG pool [34,38].

Liver, aortas, endarterectomy pieces or VSMC total RNAs were purified using TRIZOL^R ^protocol (Invitrogen, Cergy-Pontoise, France) with the addition of a DNase treatment. Concentrations and purity were verified by measuring optical density at 230, 260 and 280 nm and integrity by agarose gel electrophoresis. For measurements of individual mRNA levels, total RNA was reverse transcripted using Superscript II (Invitrogen) and random hexamers. Real time PCR was performed in a MyIQ thermal cycler (Biorad, Marnes La Coquette, France) using iQ SYBR green Supermix (Biorad). All samples were run in duplicate along with dilutions of known amounts of target sequence for quantification of initial cDNA copies. Results are expressed as the target over 18S RNA concentration ratio (ng/μg). Primer sequences are shown in table 1.

**Table 1:** Primers used for the determination by real-time PCR of mRNA concentrations:

Name	Forward primer	Reverse primer	Size (bp)
In rats			

ACC1	caacgcaggcatcagaa	caagtattccacagtccc	138

ChREBP	cgggacatgtttgatgactatgtc	aataaaggtcggatgaggatgct	86

FAS	ggtgctacccattcgtg	ggatgtatcattcttggactt	115

Srebp-1c	cgctaccgttcctctatcaa	ttcgcagggtcaggttctcc	164

VLDLr	tctggagttcctagctcat	ccagtgaatttattggcacc	108

FAT	aggaagtggcaaagaat	tgaaggctcaaagatgg	155

LPL	cctgaagacacagctgagga	cacccaactctcatacattc	141

In mice:			

ACC1	cgctggtcttagaagttga	tccctgcygatgtatttgat	149

ChREBP	gtccgatatctcgacacactc	cattgccaacataagcatgttctg	91

FAS	tgctgccgtgtccttctacca	gcacccaagtcctcgccata	128

Srebp-1c	ggcactaagtgccctcaacct	gccacatagatctctctgccagtgt	81

FATP	cctgcggcttcaaca	tcagtggctccatcgt	84

FAT	ggaactgtgggctcattgc	catgagaatgcctccaaacac	68

In humans			

ACC1	acatccctacgctaaaca	agaacatcgctgacacta	85

ChREBP	tcggcaatgctgacatg	gaggcgggagttggtaaa	98

FAS	acggccctcatttccag	tgaagctcacccagttatcc	87

Srebp-1c	tgaagacagacggagcca	ggactgttgccaagatggtt	120

18S (mice, Rat, human)	tgaggccatgattaagaggg	agtcggcatcgtttatggtc	190

### Statistics

Results are shown as mean ± sem. For in vivo rat studies comparisons were performed by two-way ANOVA (factors: time and genotype) followed by Bonferroni test. BMI and plasma values of diabetic and non-diabetic subjects were compared by two tailed t test for unpaired values and mRNA values were compared by two-way ANOVA (diabetic or not, atheroma plaque or MIT) followed by Bonferroni test. For *in vitro *studies, comparisons of data obtained from VSMC of ZO and CO rats in the initial (basal) state and after culture without or with high glucose concentration and/or ADM were performed by two-tailed Student t test for unpaired values. Data obtained in the absence or presence of insulin, T3, AngII, of one of the LXR or PXR agonists, were compared by one way ANOVA followed by the Dunnett test to locate the differences. Values obtained in mice having received GW3965 or vehicle alone were compared by two-tailed Student t test for unpaired values. P < 0.05 was considered as indicating a significant difference. Calculations were performed with GraphPad Prism 4.02 (GraphPad, San Diego, CA, USA).

## Results

### 1. Hormone and metabolites values in rats (Tables 2 and 3)

**Table 2:** Plasma values in Zucker obese (ZO) and Zucker diabetic (ZDF) rats and in their respective controls (CO and CZ).

	CZ	ZDF	CO	ZO
Triglycerides mM				
7 weeks	0.53 ± 0.06	4.12 ± 0.48***	0.49 ± 0.09	1.19 ± 0.09**
14 weeks	0.54 ± 0.07	3.78 ± 0.39***	0.55 ± 0.07	2.15 ± 0.30**
21 weeks	0.59 ± 0.05	5.85 ± 1.03***	0.72 ± 0.17	3.73 ± 1.09*$

Glucose mM				
7 weeks	7.39 ± 0.28	7.32 ± 0.31	6.89 ± 0.13	7.56 ± 0.53
14 weeks	7.02 ± 0.39	30.11 ± 0.91***	7.52 ± 0.58	7.90 ± 0.34
21 weeks	7.58 ± 0.32	30.20 ± 0.21***	6.05 ± 0.64	10.59 ± 2.45*$

Insulin μg/L				
7 weeks	4.7 ± 0.7	16.0 ± 3.0**	0.8 ± 0.4	4.8 ± 1.0**
14 weeks	6.8 ± 1.1	4.5 ± 1.0	1.7 ± 0.4	6.7 ± 0.3***
21 weeks	8.5 ± 0.5	<0.05**	1.2 ± 0.2	6.3 ± 0.5***

Measurements were made in the post-absorptive state.

* p < 0.05, ** p < 0.01, *** p < 0.001 vs the respective control group; $ p < 0.05 vs the value at 7 weeks of the same group

**Table 3:** TG content in aortas of Zucker obese (ZO) and diabetic (ZDF) rats and in their respective controls (Co and CZ).

TG μg/mg tissue	CZ	ZDF	CO	ZO
7 weeks	0.28 ± 0.06	2.10 ± 0.42**	27.8 ± 5.8	49.1 ± 5.3*
14 weeks	0.50 ± 0.09	2.57 ± 0.37**	26.4 ± 4.2	66.9 ± 7.8**
21 weeks	2.64 ± 1.16 $	4.92 ± 1.37 $	46.6 ± 11.2	66.6 ± 7.2

* p < 0.05, ** p < 0.01 vs the respective control group; $ p < 0.05 vs the value at 7 weeks of the same group

7 week old ZDF rats had normal glucose concentrations but high insulin levels (p < 0.01) indicating the presence of insulin-resistance (table 2). They had overt diabetes at the age of 14 and 21 weeks, with persistence of some insulin secretion at 14 weeks, but almost undetectable insulin level at 21 weeks. Plasma TG concentrations were very high in ZDF rats (p < 0.001 vs CZ group). TG concentrations in aortas (table 3) increased with age in both CZ and ZDF rats, with values higher in both groups at 21 than at 7 week (p < 0.05). These concentrations were higher in ZDF rats at 7 and 14 week (p < 0.01) with a trend for higher values at 21 weeks.

ZO rats were insulin-resistant at 7 and 14 weeks, with normal glucose but raised insulin levels, and developed mild type 2 diabetes at 21 weeks (table 2). Their plasma TG concentrations increased with age and were always higher than in CO rats. TG concentrations in aortas were higher in ZO and CO rats than in ZDF and the CZ rats respectively. There was a non significant trend for higher values with increasing age in both ZO and CO rats and concentrations were higher in ZO than in the corresponding control group at 14 weeks with a non-significant trend at 7 and 21 weeks (table 3).

### 2. Expression in arterial wall of genes involved in *de novo *lipogenesis and in plasma lipids uptake (figures 1 and 2)

**Figure 1:**
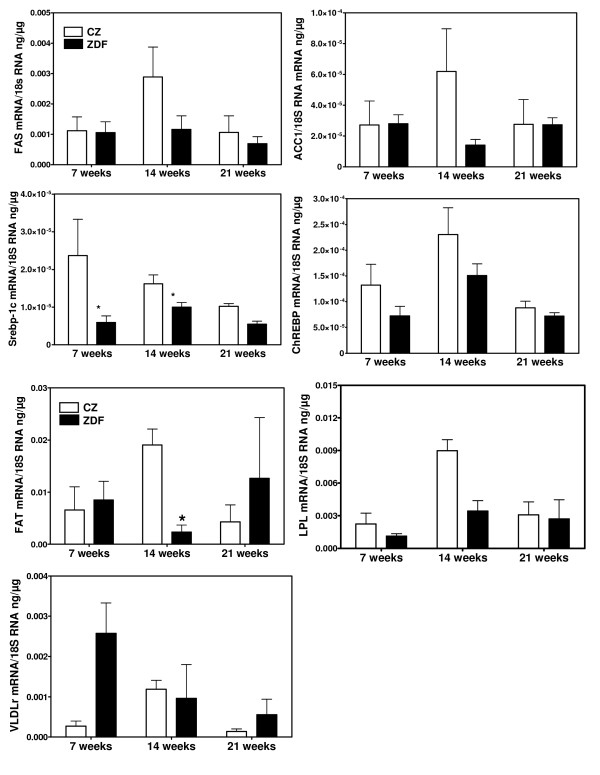
**mRNA concentrations for genes involved in fatty acid synthesis (FAS, ACC1, Srebp-1c, ChREBP) and in uptake of fatty acids from plasma lipids (FAT, LPL, VLDLr) in aortas of control (CZ) and ZDF rats studied at the age of 7, 14 and 21 weeks**. * p < 0.05, ** p < 0.01 vs the corresponding value of control rats.

**Figure 2:**
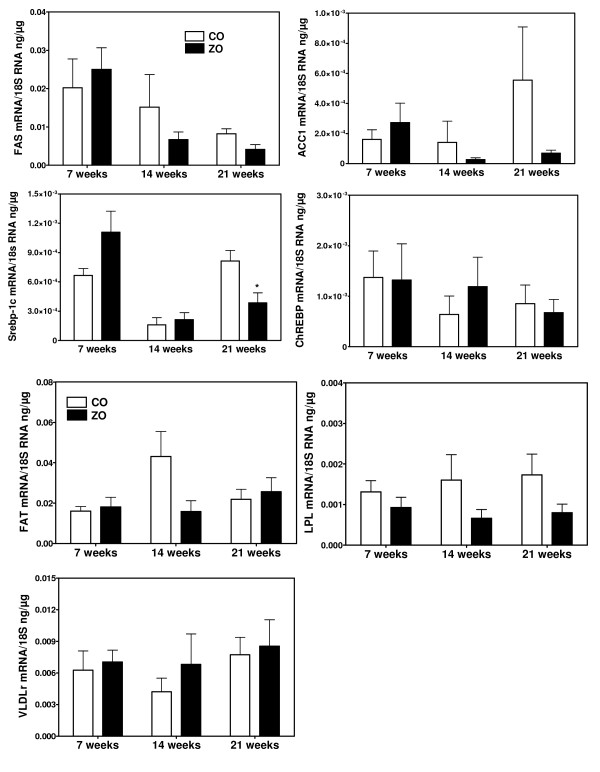
**mRNA concentrations for genes involved in fatty acid synthesis (FAS, ACC1, Srebp-1c, ChREBP) and in uptake of fatty acids from plasma lipids (FAT, LPL, VLDLr) in aortas of control (CO) and ZO rats studied at the age of 7, 14 and 21 weeks**. * p < 0.05 vs the corresponding value of control rats.

**Figure 3:**
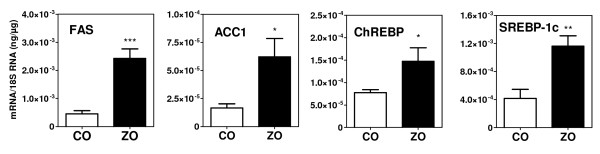
**Expression of lipogenic genes in the liver of 14 week old control (CO) and obese (ZO) Zucker rats**. * p < 0.05, ** p < 0.01, *** p < 0.001 vs values in CO rats.

We next measured in aortas of ZDF (figure 1) and ZO rats (figure 2) and of their corresponding controls the mRNA levels for key enzymes of DNL (ACC1, FAS) and for transcription factors mediating the stimulatory effects of insulin (Srebp-1c) and glucose (ChREBP) on the expression of lipogenic genes. The expression of these genes did not increase with age in both ZDF and ZO rats, as well as in their respective controls. Moreover mRNA levels were not increased either in ZDF or in ZO rats. Concentrations were comparable to those found in control rats or slightly decreased as for Srebp-1c. Therefore there was no correlation between the expression of lipogenic genes and TG content in aortas and no evidence for an overexpression in aorta of the lipogenic pathway in these models of insulin-resistance and diabetes. This lack of increased expression of lipogenic genes in aortas contrast with the increase found in liver of ZO rats (figure 3) and previously reported in livers of ZDF rats [35,39,40]. In the present experiments, the expression of genes involved in the uptake of plasma NEFA (FAT) or of fatty acids from TG rich lipoproteins (LPL and VLDLr) also did not increase with age and was not higher in aortas from ZDF and ZO rats than from their controls. Taken all together these results suggest that the increased TG content of aorta in ZO and ZDF rats does not result from an increased expression of pathways for fatty acids synthesis or uptake but probably merely of an increased availability for uptake of plasma lipid substrates.

**Figure 4:**
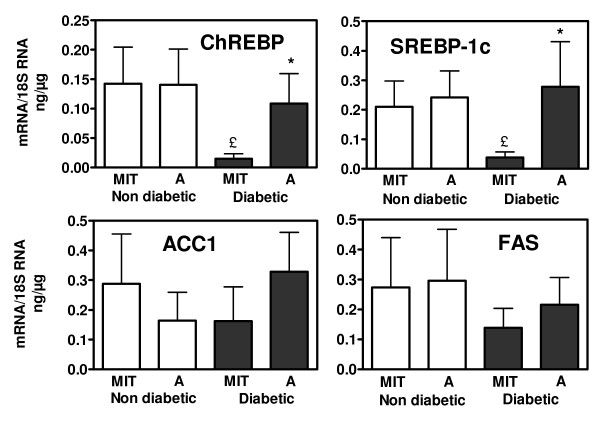
**Concentrations of mRNA for lipogenic genes measured in human atheroma plaques (A) and in adjacent macroscopically intact tissue (MIT)**. Samples were collected during carotid endarterectomy in diabetic (n = 6) or non diabetic (n = 6) subjects. * p < 0.05 vs values in the MIT of the same subjects; £ p < 0.05 vs the corresponding value of non diabetic subjects.

Lastly we measured the expression of lipogenic genes in human arterial wall by measuring relevant mRNA levels in atheroma plaques and nearby macroscopically intact tissue (MIT) collected during endarterectomy in diabetic and non diabetic subjects with carotid atheroma (figure 4). In MIT, expressions of FAS, ACC1, Srebp-1c and ChREBP were not increased but rather decreased in diabetic subjects. In non-diabetic subjects there was no difference in mRNA concentrations between MIT and atheroma whereas in diabetic patients there was in plaques a clear trend for higher values with concentrations, for ACC1, Srebp-1c and ChREBP near to those observed in plaques of non-diabetic subjects. These variations could merely reflect differences in cell population, but there is clearly in diabetic humans compared to non-diabetic subjects no increased expression of lipogenesis in arterial wall, as in diabetic rats.

### 3. Expression in cultured VSMC of genes involved in DNL and in lipids uptake

**Figure 5:**
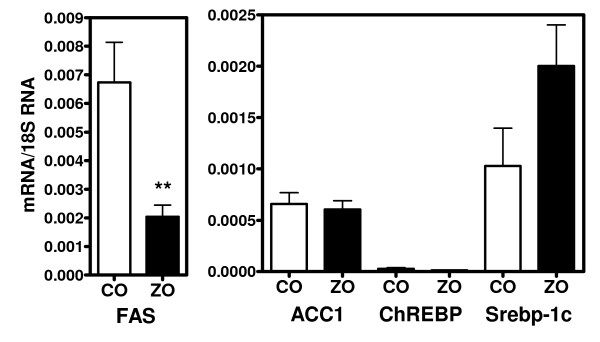
**Lipogenic mRNA concentrations measured in the basal state in cultured VSMC obtained from aortas of Zucker obese (ZO) and control (CO) rats**. ** p < 0.01 vs the value of controls.

**Figure 6:**
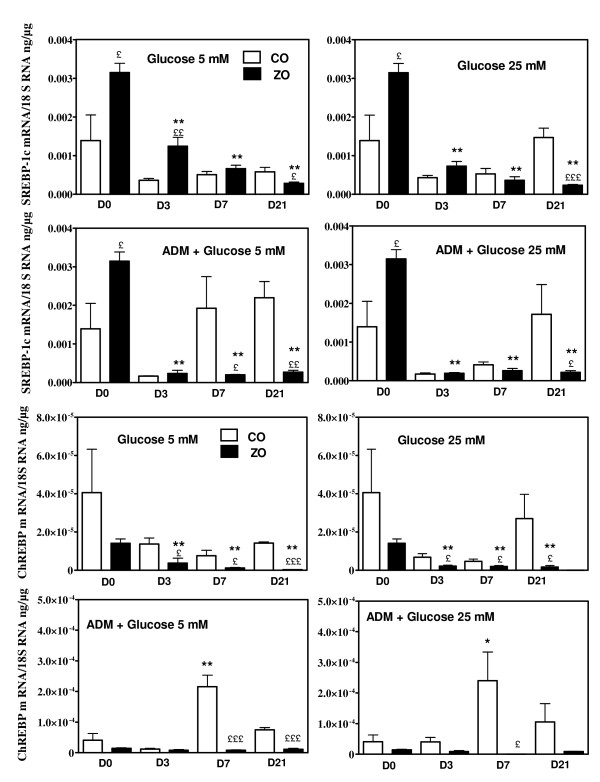
**Srebp-1c and ChREBP mRNA concentrations in cultured VSMC of Zucker obese (ZO) and control (CO) rats.** Concentrations were measured in the basal state (D0, glucose 5 mM) and after 3, 7 and 21 days of culture (D3, D7, D21) in basal conditions (glucose 5 mM), in presence of raised glucose concentration (25 mM) or in the presence of adipogenic differentiation medium (ADM) without or with raised glucose concentrations. * p < 0.05, ** p < 0.01 vs the value observed at T0; £ p < 0.05, ££ p < 0.01, £££ p < 0.001 vs the corresponding value of VSMC of control rats.

**Figure 7:**
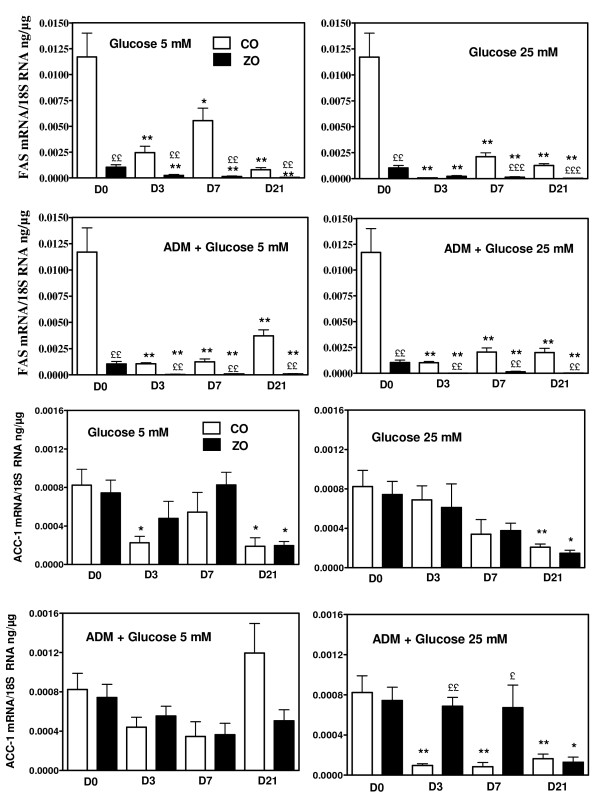
**FAS and ACC1 mRNA concentrations in cultured VSMC of Zucker obese (ZO) and control (CO) rats. **Concentrations were measured in the basal state (D0, glucose 5 mM) and after 3, 7 and 21 days of culture (D3, D7, D21) in basal conditions (glucose 5 mM), in presence of raised glucose concentration (25 mM) or in the presence of adipogenic differentiation medium (ADM) without or with raised glucose concentrations. * p < 0.05, ** p < 0.01 vs the value observed at T0; £ p < 0.05, ££ p < 0.01, £££ p < 0.001 vs the corresponding value of VSMC of control rats.

**Figure 8:**
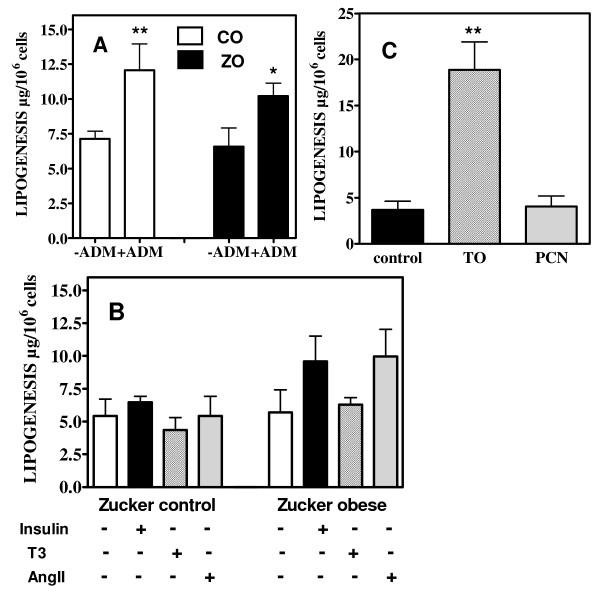
**Activity of the lipogenic pathway (expressed as contribution to the cellular TG pool) measured in VSMC of control and obese Zucker rats cultured in the presence or absence of adipogenic medium (ADM) (panel A), in the presence or absence of insulin, trioiodothyronine (T3) or angiotensin II (AngII) (panel B) and in VSMC of control Zucker rats cultured without (control) or with TO901317 (TO) or PCN (panel C)**. * p < 0.05, ** p < 0.01 vs the corresponding control situation.

**Figure 9:**
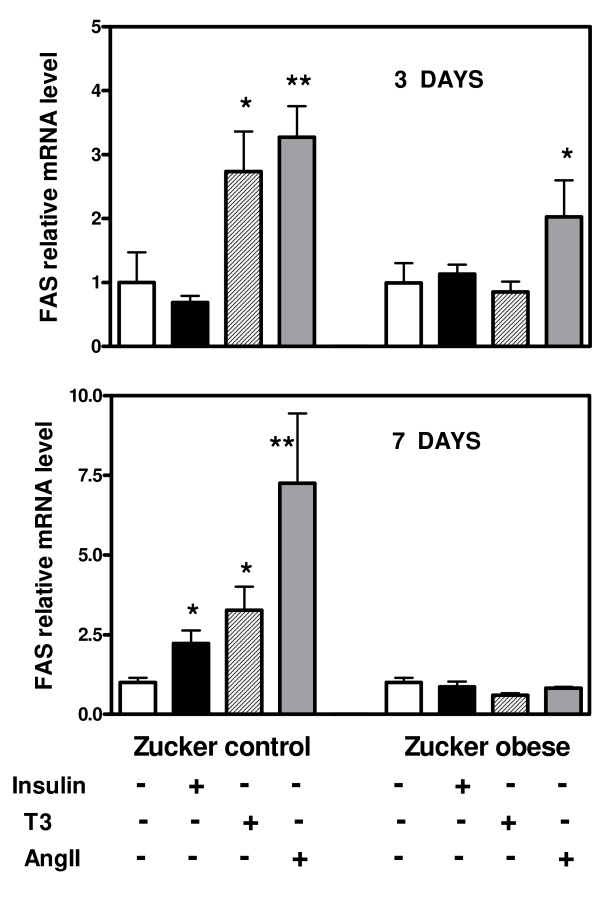
**FAS mRNA concentrations in VSMC of Zucker obese and control rats after three or seven days of culture in the absence (-) or presence (+) of insulin, T3 or angiotensin II**. * p < 0.05, ** p < 0.01 vs the corresponding value in the absence (-) of the tested molecule.

We next investigated the expression of lipogenic genes in cultured VSMC obtained from aortas of Zucker obese and control rats. mRNA concentrations of ACC1, FAS, Srebp-1c and ChREBP were measured in basal conditions (DMEM with 5 mM glucose) (D0) and then during 3, 7 and 21 days (D3, D7, D21) of culture in presence of high glucose concentrations (25 mM) and/or adipogenic medium (ADM) compared with 3, 7 and 21 days of culture in basal conditions (5 mM glucose, no ADM). In the initial state (D0, DMEM 5 mM glucose) there was a trend for higher Srebp-1c mRNA concentration in VSMC of Zucker obese rats, whereas mRNA concentrations of ChREBP and ACC1 were comparable and those of FAS decreased (p < 0.05) in cells from Zucker obese rats (figure 5). When culture was continued in basal conditions (DMEM, 5 mM glucose) there was a progressive decline of all mRNA concentrations in VSMC of both control and obese rats (figure 6 and 7). This fall in mRNA values was for Srebp-1c, ChREBP and FAS more important in cells of obese rats and at 21 days, all these values were lower in cells from obese than from control rats (figure 6 and 7). The addition of high glucose concentration or of adipogenic medium opposed in part the decline of Srebp-1c and ChREBP mRNA concentrations in VSMC of control rats, but without significant modifications in the evolution of ACC1 and FAS mRNA values. Neither high glucose concentration nor adipogenic medium modified the evolution of mRNA values in cells from Zucker obese rats, and values attained at 21 days were always lower than in cells from control rats. In addition, ADM increased largely at 21 days TG content (233 ± 22 vs 114 ± 11 μg/10^6 ^cells, p < 0.001) and the contribution of DNL to this TG pool (12.1 ± 1.9 vs 7.1 ± 0.5 μg/10^6 ^cells, p < 0.05) in VSMC from control rats whereas the increases were only moderate in cells of obese rats (170 ± 11 vs 129 ± 17 μg/10^6 ^cells, p < 0.05 and 10.1 ± 0.9 vs 6.6 ± 1.3 μg/10^6 ^cells p < 0.05, respectively) (figure 8). Insulin or T3 alone increased moderately FAS mRNA, always only in cells from control rats (figure 9), but not other lipogenic mRNA concentrations (data not shown). In addition, neither the cell content of TG nor the activity of the lipogenic pathway measured with deuterated water were modified by insulin or T3 in cells from control or obese rats (figure 8). AngII had a marked effect on FAS expression in VSMC of control rats and a moderate action in cells from obese rats (figure 9). However other lipogenic mRNAs were not significantly modified by AngII and cellular TG content as well as activity of lipogenesis (figure 8) were unchanged.

**Figure 10:**
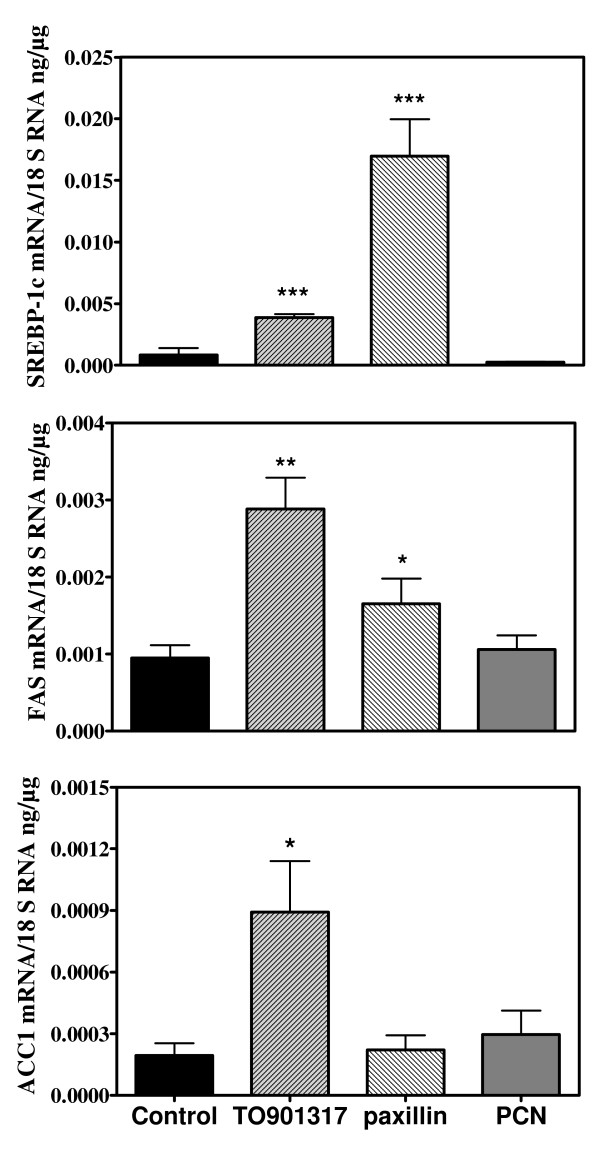
**Response of lipogenic genes to agonists of PXR (PCN), LXR (paxillin) and to a dual PXR and LXR agonist (TO901317). mRNA concentrations (VSMC of control rats) were measured after three days of culture (glucose 5 mM, no ADM) in the absence (control) or presence of the various agonists**. * p < 0.05, ** p < 0.01, ***p < 0.001 vs control.

**Figure 11:**
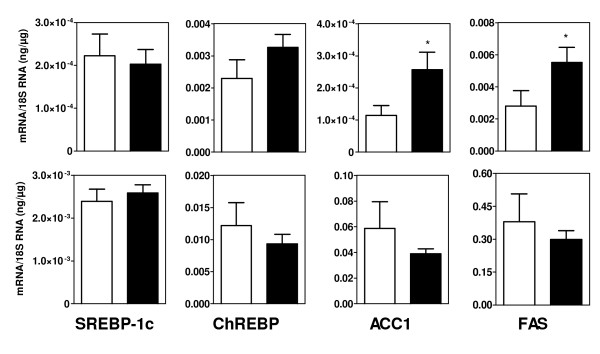
**Expression of lipogenic genes in liver (upper panel) and aortas (lower panel) of mice after a three days administration of GW3965 (black columns) or vehicle alone (control, white columns)**. * p < 0.05 vs the control group.

Davies et al reported that TO901317 stimulated the expression of lipogenic genes in VSMC and ascribed it to a stimulation of LXRα [13]. However TO901317 is a dual LXR and PXR agonist [27]. PXR stimulates lipogenesis in liver [28], is expressed in vasculature [29] and we found that it is expressed at least at the mRNA level in VSMC (data not shown). Therefore we compared the effects of TO901317 with those of paxillin (LXR agonist [28,33]) and of PCN (PXR agonist) on VSMC of control rats. Three days of culture with TO901317 increased largely TG content (from 113 ± 12 to 277 ± 23 μg/10^6 ^cells, p < 0.01). Figure 10 shows that TO901317 increased indeed the mRNA concentrations of Srebp-1c, FAS and ACC1 while PCN had no effects. Paxillin stimulated the expression of Srebp-1c and FAS but had no effect on ACC1. In addition, TO90137 increased largely the contribution of de novo lipogenesis to cell TG content, expressed as percent (6.8 ± 0.8 vs 2.7 ± 0.7%, p < 0.01) or absolute value (18.9 ± 3.0 vs 2.8 ± 0.5 μg/10^6 ^cells, p < 0.01, figure 8) while PCN had no effect (absolute lipogenesis: 4.0 ± 1.1 μg/10^6 ^cells). Taken all together these results show that the effects of TO901317 on lipogenesis are indeed mediated through activation of LXR while PXR activation has no stimulatory effect on lipogenic genes expression or on lipogenic activity in VSMC. In addition, TO901317 and paxillin had a moderate effect on the expression of FAT while PCN had no consistent action (data not shown). Since these *in vitro *results confirmed that LXR agonists stimulate the expression of lipogenic genes in VSMC we tested whether this action was detectable in arterial walls *in vivo*. Administration to mice of GW3965, a LXR agonist without action on PXR [27], increased as expected hepatic TG content (7.35 ± 1.31 vs 2.34 ± 0.56 μg/mg liver p < 0.01) and the expression of FAS and ACC1 in liver (figure 11). However SREBP-1c expression was unchanged and the increase in the mRNA level of ChREBP, considered as a target of LXR [41] was of borderline significance (p = 0.09). Liver FAT and FATP mRNA levels were not increased (data not shown). Plasma TG levels were not increased by GW3965, in agreement with previous data [27,42,43] showing that this specific LXR agonist has no significant or only a weak hypertriglyceridemic action. TG content in aortas was indeed increased by GW3965 (34.7 ± 7.5 vs 16.7 ± 2.4 μg/mg tissue p < 0.05) but without increase in the expression of lipogenic genes (figure 11). FAT mRNA was not increased either but FATP expression was stimulated (p < 0.01) suggesting that the rise in TG content could be related to an increased uptake of fatty acids.

## Discussion

The present results confirm that the TG content of aortas increases with age [13]. They show that this content is also increased in two experimental models of insulin-resistance and diabetes. The presence in these pathological situations of excessive TG accumulation in non-adipose tissues, previously reported in liver [35,44], skeletal muscle [45,46], endocrine pancreas [47] and heart [48,49], is thus extended to arterial wall. Our data also extend the previous finding that lipogenic genes are expressed in arterial wall and VSMC [13] by showing that ACC1 and ChREBP, the transcription factor mediating the stimulatory effects of glucose on lipogenic genes expression, are also expressed in this tissue and these cells.

Our first aim was to determine whether modifications of the *in situ *expression of lipogenic genes could contribute to physiological (aging) or pathological (insulin-resistance and diabetes) increases in arterial wall TG content. We found no increase with age or insulin-resistance in any of the lipogenic mRNA measured. Although we determined only mRNA concentrations, it is thus unlikely that enhanced lipogenesis contributed to the increased TG content. We also found no increase with age or insulin-resistance in the expression of genes involved in the uptake of plasma NEFA or of fatty acids of TG-rich lipoproteins. Altogether, these results suggest that the increased TG content of arterial wall observed in insulin-resistance and diabetes result mainly from the increased concentration and availability of circulating lipid substrates for uptake by cells of the arterial wall.

The lack of increase of lipogenic mRNA levels in aortas of insulin-resistant or diabetic Zucker rats strongly suggests in addition that in these situations lipogenesis is resistant to the actions of insulin and glucose. Interestingly, when measuring lipogenic mRNA concentrations in carotid endarterectomy samples from diabetic and non-diabetic patients, we found that theses concentrations were decreased in macroscopically intact arterial tissue of diabetic patients and comparable in atheroma plaques of diabetic and non-diabetic patients, supporting also the presence of resistance to insulin of arterial lipogenesis in human diabetes. Data obtained in cultured VSMC of ZO rats also support this resistance. In basal conditions, FAS expression was decreased. More importantly, only cells of control rats had a moderate response of lipogenic genes to glucose and adipogenic factors in combination or alone. VSMC from ZO rats did not respond and had mRNA values largely lower than cells from control rats. Lastly the stimulatory action of ADM on the activity of the lipogenic pathway was decreased in VSMC from ZO rats. Taken altogether, theses results obtained in human beings, in rats and in cultured VSMC strongly support the idea that the insulin-resistance of arterial wall previously described [9] involves the lipogenic pathway. This resistance could be more general since the stimulatory effects of T3 and AngII on FAS expression were also reduced or abolished in cells from ZO rats.

The status of arterial wall lipogenesis in situations of insulin-resistance is thus comparable to the one observed in skeletal muscle [26] and adipose tissue [50]: decreased basal expression and resistance to the action of insulin. This repression of lipogenesis contrasts sharply with its hepatic overexpression in both experimental models of insulin-resistance [35,44] and human subjects with obesity and insulin-resistance [38,50]. In addition, this overexpression of liver lipogenic genes contributes to the excessive hepatic accumulation of TG found in these situations [25,38,51,52]. The reasons behind this discrepancy in the status of lipogenesis between liver on one hand and adipose tissue, skeletal muscle and VSMC on the other remain unclear. Whatever the reasons for this discrepancy, the increased lipid accumulation found in arterial wall of insulin-resistant and diabetic rats could contribute to its insulin-resistance [9]

Lastly, we confirm the previously described stimulatory effect of TO901317 on expression of lipogenic genes [13] in VSMC. We show in addition that this is accompanied by a clear increase in the activity of the lipogenic pathway and is mediated though activation of the nuclear receptor LXR since this effect is reproduced by a specific LXR agonist but not by a selective PXR agonist. LXR agonists have been proposed as a possible treatment of atheroma. Indeed they reduce the development of atheroma in mice model [53] through stimulation of cellular cholesterol efflux and reverse transport [54], and possibly also through some anti-inflammatory action [55]. A drawback with compounds such as TO931317 is the rise in plasma TG concentrations. This could be solved by using other, more specific, LXR agonists such as GW3965. Indeed, in agreement with previous reports [27,42,43], GW3965 did not increase plasma TG levels in mice. However, despite the lack of significant increase of the expression of lipogenic genes in arterial wall, GW3965 increased in the present report arterial TG content. The possible deleterious effects on a long term basis of this increase in arterial lipids accumulation should be kept in mind. Clarifying the consequences of such direct actions of LXR agonists on arterial wall on the development of atheroma will require further studies.

In summary, we found that arterial wall TG content increases with age and in situations of insulino-resistance and type 2 diabetes. Lipogenic genes are expressed in normal and pathological (atheroma plaques) arterial wall as well as in VSMC. Their expression is stimulated *in vitro *by glucose, ADM and LXR agonists. However, these expressions are not increased during insulin-resistance and diabetes and resist to the *in vitro *actions of glucose and ADM. Therefore, it is unlikely that *in *situ (arterial wall) lipogenesis contributes to lipid accumulation in arterial wall during insulin-resistance and diabetes and thus to the increased risk of atheroma observed in these situations.

## Competing interests

The authors declare that they have no competing interests.

## Authors' contributions

NH realized part of the in vivo protocols and most of the in vitro studies and measurements of mRNA, and drafted the paper. FF was involved in vitro studies and set up most of mRNA quantifications. SN participated in the in vitro studies and the in vivo study in mice. PdC participated to in vivo protocols in rats and mice. PF and GB were responsible for the studies in humans. SAB and MB conceived the study, were responsible for the general design and coordination and drafted the paper. All authors approved the final manuscript.
